# Exploring the effects of lysozyme dietary supplementation on laying hens: performance, egg quality, and immune response

**DOI:** 10.3389/fvets.2023.1273372

**Published:** 2023-10-06

**Authors:** Daniel Sindaye, Zaili Xiao, Chaoyu Wen, Kang Yang, Limeng Zhang, Pinfeng Liao, Fan Zhang, Zhongquan Xin, Shansong He, Shibin Ye, Dan Guo, Suqin Hang, Shehata Zeid, Baichuan Deng

**Affiliations:** ^1^Maoming Branch, Guangdong Laboratory for Lingnan Modern Agriculture, Guangdong Provincial Key Laboratory of Animal Nutrition Control, National Engineering Research Center for Breeding Swine Industry, College of Animal Science, South China Agricultural University, Guangzhou, China; ^2^Jiangsu Key Laboratory of Gastrointestinal Nutrition and Animal Health, Laboratory of Gastrointestinal Microbiology, National Center for International Research on Animal Gut Nutrition, Nanjing Agricultural University, Nanjing, China

**Keywords:** lysozyme, laying performance, biochemical indices, egg quality, body immunity, intestinal morphology

## Abstract

An experiment was conducted to evaluate the dietary supplementation with lysozyme's impacts on laying performance, egg quality, biochemical analysis, body immunity, and intestinal morphology. A total of 720 Jingfen No. 1 laying hens (53 weeks old) were randomly assigned into five groups, with six replicates in each group and 24 hens per replicate. The basal diet was administered to the laying hens in the control group, and it was supplemented with 100, 200, 300, or 400 mg/kg of lysozyme (purity of 10% and an enzyme activity of 3,110 U/mg) for other groups. The preliminary observation of the laying rate lasted for 4 weeks, and the experimental period lasted for 8 weeks. The findings demonstrated that lysozyme might enhance production performance by lowering the rate of sand-shelled eggs (*P* < 0.05), particularly 200 and 300 mg/kg compared with the control group. Lysozyme did not show any negative effect on egg quality or the health of laying hens (*P* > 0.05). Lysozyme administration in the diet could improve intestinal morphology, immune efficiency, and nutritional digestibility in laying hens when compared with the control group (*P* < 0.05). These observations showed that lysozyme is safe to use as a feed supplement for the production of laying hens. Dietary supplementation with 200 to 300 mg/kg lysozyme should be suggested to farmers as a proper level of feed additive in laying hens breeding.

## 1. Introduction

For more than half a century, antibiotics have played an important role in improving poultry production efficiency (1, 2). Despite their benefits, their usage has shown side effects on human health, which were gradually appearing (3). The poultry industry places a high priority on the hunt for antibiotic alternatives due to their restriction in several nations (4). These alternatives include probiotics (5, 6), prebiotics (7), synbiotics (8, 9), plant extracts (10), organic acids (11), essential oils, and exogenous enzymes (12–14). Among the exogenous enzymes, lysozyme has already been used in different domains including medicine and food, the reason why it has attracted the attention of the feed industry. Lysozyme (1,4-N-acetylmuramidase) works primarily against gram-positive bacteria (15) by cleaving the 1,4-glycosidic bond between the N-acetylmuramic acid and N-acetylglucosamine residues of bacterial peptidoglycan, resulting in cell lysis (15, 16).

Lysozyme is an antibacterial enzyme that is extremely resistant to digestion in the gastrointestinal tract and can be hydrolyzed by acids and proteases (17, 18). The effectiveness of lysozyme as an antibiotic substitute in pig and broiler feed has been demonstrated (19, 20). The administration of lysozyme increased the average daily weight gain (ADG) and enhanced the feed conversion ratio of broiler chickens (21). In the previous research in weaned pigs, 90 mg/kg lysozyme contributed to greater ADG than antibiotic-treated (22). Moreover, it has been shown that lysozyme reduced the pathogen levels in the cecum of broilers (17, 23) while increasing the villus height and villus/crypt ratio of the duodenum (22). Nevertheless, there are little data on the benefits of lysozyme supplementation in laying hens' diet, particularly at the late stage of development of laying hens. Therefore, our current research aimed to analyze the dietary supplementation with lysozyme's effects on production, egg quality, biochemical analysis, antioxidant status, immunological parameters, and intestinal morphology of laying hens.

## 2. Materials and methods

### 2.1. Animal ethics

This research, involving animals, was performed with respect to the South China Agricultural University's sanitary protocol on animal ethics during sample collection. All steps were followed to prevent laying hens from suffering during this research, according to South China Agricultural University's Animal Ethics Commission advice in compliance with the Chinese Animal Welfare Guidelines (Animal Ethic number 2020g015).

### 2.2. Experiment plan, diets, and management

A total of 720 Jingfen No. 1 laying hens with no significant difference in laying performance (53 weeks old) were randomly assigned into 5 groups, with 6 replicates per treatment and 24 hens per replicate (144 laying hens/treatment). These groups were the control group, L100, L200, L300, and L400. The laying hens in the control group were fed with the basal diet, and for the 4 other groups, the basal diet was supplemented with 100, 200, 300, or 400 mg/kg of lysozyme, respectively. The lysozyme provided in the L100, L200, L300, or L400 groups was the same and had 10% purity and 3,110 U/mg enzyme activity. The feedstuff ingredients used and their percent in the basal diet are shown in Table 1.

**Table 1:** Composition and nutrient levels of the basal diet (%, as air-dry basis).

**Ingredients**	**Content**	**Nutrient levels^b^**	**Content**
Corn	55.50	ME/(Kcal/kg)	2,598.65
Soybean meal	26.00	Crude Protein	16.30
Limestone	10.85	Calcium	4.47
Biological protein	3.00	Total Phosphorus	0.64
Soybean oil	2.30	Assimilable Phosphorus	0.42
CaHPO_4_	1.35	Methionine	0.41
Premix^a^	1.00	Lysine	0.96
Total	100.00		

a The premix provided the following per kg of the diets: VA: 14,000 IU, VD3:7,000 IU, VE: 71 IU, VK3: 2.625 mg, VB1: 3.5 mg, VB2: 10.5 mg, VB6: 5.25 mg, VB12: 0.02 mg, VC: 80 mg, folic acid: 1.05 mg, biotin: 0.07 mg, niacin: 35 mg, D-pantothenic acid: 15.75 mg, Fe: 80 mg, Cu: 8 mg, Mn: 100 mg, Zn: 60 mg, Co: 0.2 mg, I: 0.5 mg, Se: 0.4 mg, 50% Choline chloride: 1.05 g, L-Lys: 1.36 g, DL-Met: 1.71g, NaHCO3: 1 g, NaCl : 3 g and De-mold agent: 4 g.

b The nutrient levels were calculated values.

The preliminary test lasted for 4 weeks, and the formal period lasted for 8 weeks. During the preliminary test period, the laying hens were fed with the basal diet, and the laying performance was observed to see that there was no significant difference in laying performance before the administration of lysozyme as a feed additive. Laying hens were maintained in semi-opened enclosures within 3-layer complete ladder cages containing 4 hens per cage. The hens had *ad libitum* access to feed and water throughout the study period. The average daily temperature was 26±5°C, with a photo-period of 16L:8D (16 h of 1ighting and 8 h for obscurity).

### 2.3. Production performance

The total egg number, egg mass, and unqualified (sand-shelled, soft, and broken) egg numbers were recorded daily during the experiment. The average feed intake (AFI) was quantified weekly in each replicate, and the production performance parameters were calculated as follows: Daily egg production rate of laying hens (%) = total number of eggs produced during the statistical period/(number of laying hens × number of days) × 100%; Average daily egg weight = total egg weight in the statistical period (kg)/statistical days; Average daily feed intake = (total feed intake) (kg)/(number of laying hens × number of days); Feed conversion rate (FCR) = feed consumption in the statistical period (kg)/total egg weight in the statistical period (kg); Average egg weight = total egg weight during the statistical period (g)/total number of eggs during the statistical period; Soft and broken egg rate (%) = number of soft and broken eggs/total number of eggs × 100%; Sand-shelled egg rate (%) = Sand-shelled egg rate/total number of eggs × 100%.

### 2.4. Egg quality

Egg samples were taken on the last day of the 4th week and 8th week of this experiment. In total, 5 eggs were randomly taken in each replicate for 30 eggs in each group and 150 eggs in the 4th week and 150 eggs in the 8th week (300 eggs were used as samples in this current experiment). A Vernier caliper was used to measure the length and width of eggs (egg shape index= length/width). The measure of eggshell breaking strength was performed by an Eggshell Strength Tester (NFN388, FHK, Japan). An egg multi-tester was used to determine the albumin height, Haugh unit, and egg yolk color (EMT 5200, Robotmation Co., Ltd.). Using an eggshell thickness gauge, the determination of eggshell thickness was measured by the average of the blunt end, tip end, and equatorial region on an eggshell without a membrane (Robotmation Co., Ltd.).

### 2.5. Plasma biochemical indices, antioxidant status, and immunity index

At the end of this experiment, two healthy laying hens per replicate (n=12/treatment) were randomly selected and fasted for 24 h. In total, 5 ml of blood was collected from the wing veins using a heparin sodium anticoagulant tube, and the blood samples were centrifugated for 15 min at 3,000 rpm and 4°C to obtain plasma samples. An automatic biochemical analyzer (Chemray 800) was used to measure plasma biochemical parameters, such as alanine aminotransferase (ALT), aspartate aminotransferase (AST), albumin (ALB), total protein (TP), blood urea nitrogen (BUN), uric acid (UA), triglycerides (TG), total cholesterol (TC), calcium (Ca), and phosphorus (P).

Commercial kits (Nanjing Jiancheng Bioengineering Institute, Nanjing, Jiangsu, China) were used for analyzing the plasma antioxidant status. The parameters analyzed included superoxide dismutase (SOD), malondialdehyde (MDA), glutathione peroxidase (GSH-Px), catalase (CAT), and total antioxidant capacity (T-AOC). The immunological parameters such as interferon [IFN- γ, interleukins 4 (IL-4), 2(IL-2), and 6 (IL-6), immunoglobulins A (IgA), G (IgG), and M (IgM), and tumor necrosis factor α (TNF-α) concentrations in plasma were tested using chicken-specific ELISA kits (Jiangsu Meimian Industrial Co., Ltd, Yancheng, Jiangsu, China].

### 2.6. Intestinal morphology

For each replicate, 1 laying hen was selected (n=6/treatment) and slaughtered by cervical dislocation, and the entire length of the intestine was removed for further analysis. In total, 2 cm of the middle duodenum, jejunum, or ileum were taken and rinsed carefully with 0.9% NaCl several times to eliminate the digest contents. These samples were placed in 4% paraformaldehyde for histological studies. After 24-h fixation in 4% paraformaldehyde, intestinal segments were hydrolyzed, cleaned up, covered with paraffin, and separated before being stained with eosin and hematoxylin. The morphometric variables including villus height and crypt depth were measured with OLYMPUS cellSens Standard 1.18 (Build 16686), and each slice had four intact intestinal villi chosen at random.

### 2.7. Apparent nutrient retention

During the final week of the experiment, one cage per replicate (four laying hens inside) was chosen at random, and a plastic tray was placed under it for feces collection. The feed intake and the fecal excreta (after taking out the feather) were quantified for the following 3 days. In total, 100 g of homogenized excreta sample from each cage was collected once a day for 3 days. Every sample obtained received 10 ml of hydrochloric acid (10% HCl concentration) and was kept immediately in the freezer at −20°C. The feces samples from the same cage during three successive days were put together and mixed until homogenization. These samples were dried for 48 h at 65°C in the oven and then were put for equilibration to atmospheric conditions for 24 h. After drying the feces, the weight of the excreta samples was measured before being ground through a 0.45-mm screen. The crude protein (988.05, AOAC method), dry matter (934.01, AOAC method), gross energy (IKA Calorimeter System C 5010; IKA Works, Staufen, Germany), and ash content (942.05, AOAC method) were measured. The expression of organic matter was calculated by the subtraction of the samples' weight and their weight loss after ashing. Acid-insoluble ash (Vogtmann et al., 1975) was used as an indicator for apparent nutrient retention. The apparent digestibility was calculated by using the following formula: Digestibility = 100(1-$\frac{\%AIA\;feed}{\%AIA\;fecal}$ x $\frac{\%fecal\;nutrient}{\%feed\;nutrient}$) where AIA is acid-insoluble ash.

### 2.8. Statistical analysis

Statistical analysis was made using the IBM SPSS 25 software package with one-way ANOVA as a completely randomized design. The significant difference among the treatments was determined by Duncan's test at a *P* < 0.05. The effect of supplementation levels of lysozyme was made using the orthogonal polynomials for linear and quadratic effects. All declarations of significance depended on a *P* < 0.05.

## 3. Results

### 3.1. Laying hens' production performance

Evaluation of laying hens' production performance depends on many parameters that farmers must take into consideration as they directly affect their benefits. The impact on the production performance of lysozyme as a supplement to laying hens' diet is shown in Table 2.

**Table 2:** Effect of dietary supplementation with lysosome on laying hens' production performance.

**Item**	**Level of lysozyme**					**SEM**	***P*** **-value**		
	**0**	**100**	**200**	**300**	**400**		**ANOVA**	**Linear**	**Quadratic**
**1–4 week**									
Laying rate (%)	86.26	85.57	84.65	86.88	85.77	0.48	0.699	0.926	0.855
Egg mass (g/bird/d)	51.89	51.96	51.22	52.81	51.84	0.36	0.757	0.772	0.956
Sand-shelled egg rate (%)	21.95	16.84	15.78	14.72	17.98	1.06	0.235	0.182	0.062
Soft and broken egg rate (%)	0.50	0.57	0.75	0.35	0.70	0.07	0.353	0.712	0.934
Feed intake (g/bird/d)	105.74	106.03	105.21	106.49	105.80	0.29	0.747	0.786	0.963
FCR	2.04	2.04	2.06	2.02	2.04	0.01	0.910	0.837	0.979
Egg weight (g)	60.14	60.72	60.51	60.78	60.44	0.18	0.818	0.611	0.585
**5–8 week**									
Laying rate (%)	86.64	87.91	85.87	87.12	88.43	0.46	0.444	0.400	0.486
Egg mass (g/bird/d)	52.74	53.98	52.46	53.57	54.13	0.33	0.407	0.315	0.550
Sand-shelled egg rate (%)	20.90^a^	14.54^b^	11.76^b^	11.75^b^	15.24^b^	0.96	0.007	0.035	0.001
Soft and broken egg rate (%)	0.79	0.56	0.49	0.70	0.92	0.06	0.214	0.380	0.056
Feed intake (g/bird/d)	106.06	107.17	106.74	107.55	106.64	0.31	0.638	0.489	0.446
FCR	2.01	1.99	2.04	2.01	1.97	0.01	0.633	0.566	0.580
Egg weight (g)	60.87	61.43	61.08	61.48	61.21	0.27	0.795	0.550	0.686

FCR, feed conversion ratio.

a, b Mean that there were significant difference between groups.

As shown in Table 2, dietary supplementation with lysozyme did not influence the laying rate, egg mass, soft and broken egg rate, feed intake, FCR, and egg weight (*P* > 0.05). However, when laying hens were fed with dietary lysozyme at levels of 100–400 mg/kg during the 5–8th week, the sand-shelled egg rate was significantly lower (*P* < 0.05) than the control group. The trend analysis indicates that both linear (*P* = 0.035) and quadratic (*P* = 0.007) effects were observed in this index with an increase in inclusion level of dietary lysozyme, in which 200 and 300 mg/kg of dose groups recorded the lowest sand-shelled egg rate.

### 3.2. Egg quality

Egg quality of an egg depends on its structure and composition. When laying hens' diet was supplemented with lysozyme, the characteristics of egg quality parameters are presented in Table 3.

**Table 3:** Effect of dietary supplementation lysozyme on egg quality.

**Item**	**Level of lysozyme**					**SEM**	***P*** **-value**		
	**0**	**100**	**200**	**300**	**400**		**ANOVA**	**Linear**	**Quadratic**
**4**^th^ **week**									
Egg shape index	1.32	1.31	1.34	1.32	1.32	0.00	0.254	0.818	0.673
Eggshell strength (kgf)	3.83	4.01	3.99	3.95	3.96	0.06	0.900	0.668	0.622
Egg white height (mm)	5.33	5.81	5.55	5.74	5.70	0.09	0.428	0.277	0.391
Yolk color score	10.57	10.72	10.79	10.62	10.90	0.04	0.081	0.052	0.152
Haugh unit	71.44	75.19	73.38	74.57	74.79	0.75	0.509	0.244	0.410
Eggshell thickness (μm)	337.39	333.93	340.63	340.44	332.71	1.81	0.531	0.765	0.452
**8**^th^ **week**									
Egg shape index	1.32	1.32	1.33	1.32	1.31	0.00	0.745	0.458	0.735
Eggshell strength (kgf)	3.56	3.67	3.63	3.39	3.49	0.07	0.772	0.591	0.845
Egg white height (mm)	5.51	5.92	5.61	5.66	5.97	0.10	0.510	0.358	0.626
Yolk color score	10.07	10.52	10.34	10.11	10.48	0.07	0.094	0.371	0.615
Haugh unit	69.16	74.34	71.20	72.28	74.22	0.95	0.371	0.224	0.469
Eggshell thickness (μm)	330.43	334.88	337.24	328.16	330.87	1.99	0.620	0.717	0.541

^a, b^ Mean that there were significant difference between groups.

The results of Table 3 showed that there was no significant difference in dietary supplementation with lysozyme on egg shape index, eggshell strength, yolk color score, Haugh unit, and eggshell thickness compared with the control group (*P* > 0.05). However, dietary supplementation with lysozyme did not affect the characteristics of egg quality parameters during the laying phase.

### 3.3. Serum biochemical indices and antioxidant status

Maintaining the laying hen's health in good status is very important because it ameliorates their protection against damage and increases laying performance. The results of hens' serum biochemical indices and their antioxidant status, when they were fed with a diet supplemented with lysozyme, are shown in Table 4.

**Table 4:** Effect of dietary supplementation with lysozyme on serum biochemical indices and antioxidant status.

**Item**	**Level of lysozyme**					**SEM**	***p*** **-value**		
	**0**	**100**	**200**	**300**	**400**		**ANOVA**	**Linear**	**Quadratic**
**Biochemical index**									
ALT (U/L)	13.41	13.45	14.54	14.66	14.14	0.46	0.869	0.520	0.754
AST (U/L)	233.51	216.24	219.60	232.16	253.35	5.01	0.143	0.148	0.028
ALB (g/L)	27.90	29.06	28.59	30.16	30.49	0.54	0.533	0.102	0.265
TP (g/L)	45.48	46.94	44.71	48.32	47.41	0.72	0.519	0.326	0.607
BUN (mg/dl)	1.81	2.19	1.38	1.71	1.81	0.11	0.228	0.462	0.574
UA (μmol/L)	180.84	156.27	162.43	163.54	187.97	5.08	0.229	0.382	0.080
TG (mmol/L)	13.11	13.66	15.01	13.75	13.25	0.62	0.891	0.860	0.613
TC (mmol/L)	2.96	2.91	3.23	2.82	2.86	0.13	0.883	0.847	0.824
Ca (mmol/l)	3.35	3.45	3.32	3.46	3.41	4.00	0.779	0.623	0.875
P (mmol/l)	1.95	2.19	2.06	2.07	1.81	0.06	0.350	0.401	0.141
**Antioxidant status**									
SOD(U/mL)	385.08	398.06	383.51	424.79	417.31	9.80	0.586	0.214	0.454
MDA (nmol/mL)	5.30	5.60	5.14	4.83	4.94	0.28	0.925	0.488	0.781
GSH-Px (μmol/L)	972.71	1,322.00	987.08	1,307.96	1,475.95	81.94	0.205	0.107	0.241
CAT (U/mL)	1.08	1.06	1.56	2.13	1.13	0.24	0.262	0.523	0.540
T-AOC (mM/mL)	0.56	0.49	0.47	0.63	0.65	0.04	0.511	0.298	0.298

ALT, alanine aminotransferase; AST, aspartate aminotransferase; ALB, albumin; TP, Total Protein; BUN, blood urea nitrogen; UA, Uric acid; TG, Triglycerides; TC, total cholesterol; Ca, Calcium; P, phosphor; SOD, superoxide dismutase; MDA, malondialdehyde; GSH-Px, glutathione peroxidase; CAT, catalase; T-AOC: total antioxidant capacity.

^a, b^ Mean that there were significant difference between groups.

The results of Table 4 showed that there were no significant differences in dietary supplementation with lysozyme on ALT, ALB, TP, UN, UA, TG, TC, Ca, and P in the plasma of laying hens compared with the control group (*P* > 0.05). Similarly, dietary supplementation with lysozyme did not significantly influence laying hens' antioxidant status parameters (*P* > 0.05). As dietary lysozyme levels increased, AST levels decreased quadratically (*P* = 0.028); the lowest AST levels were recorded with the 100 and 200 mg/kg lysozyme.

### 3.4. Plasma immune parameters

As laying hens' immunity is very important for their resistance to diseases, in this current research, the parameters of laying hens' immunity were analyzed. Table 5 presents the results of laying hens' immunity when their diet was supplemented with lysozyme.

**Table 5:** Effect of dietary supplementation with lysozyme on immunity index of laying hen.

**Item**	**Level of lysozyme**					**SEM**	***p*** **-value**		
	**0**	**100**	**200**	**300**	**400**		**ANOVA**	**Linear**	**Quadratic**
IFN-γ (pg/mL)	128.03	127.56	129.36	126.81	123.47	0.95	0.374	0.143	0.152
IL-4 (ng/L)	157.28	164.23	168.37	153.65	159.55	1.74	0.058	0.627	0.318
IL-2 (ng/L)	177.74^a^	171.44^ab^	171.78^ab^	175.14^a^	161.82^b^	1.62	0.020	0.013	0.033
IL-6 (ng/L)	47.91	45.82	45.64	47.66	46.36	0.37	0.166	0.631	0.428
IgA (ng/mL)	7,323.06	7,036.70	6,952.92	7,225.34	6,985.52	87.21	0.616	0.435	0.614
IgG (μg/mL)	93.91^ab^	88.39^c^	93.63^ab^	97.28^a^	92.48^bc^	0.74	0.003	0.253	0.524
IgM (ng/mL)	4,584.61^ab^	4,310.37^b^	4,701.51^a^	4,569.48^ab^	4,242.32^b^	54.77	0.031	0.276	0.216
TNF-α(ng/L)	69.73	71.67	72.70	71.11	74.45	0.65	0.203	0.052	0.154

IFN-γ, interferon-gamma; IL-4, interleukin 4; IL-2, interleukin 2; IL-6, interleukin 6; IgA, immunoglobulin A; IgG, immunoglobulin G; IgM, immunoglobulin M, TNF-α, tumor necrosis factor α.

a, b Mean that there were significant difference between groups.

As shown in Table 5, dietary supplementation with lysozyme did not change the level of IFN-γ, IL-4, IL-6, IgA, IgG, IgM, and TNF-α in plasma compared with the control group (both linear and quadratic *P* > 0.05). The level of IL-2 (linear effect, *P* = 0.013; quadratic effect, *P* = 0.033) in the 400 mg/kg lysozyme group was significantly decreased compared with the control group (*P* < 0.05). The highest levels of IgG and IgM were noted with 300 mg/kg and 200 mg/kg lysozyme, respectively, in the plasma of laying hens, but there was no significant effect compared with the control group.

### 3.5. Intestinal morphology

Intestinal morphology plays an essential role in laying hens' life by influencing the gut microbiota. Then, its assessment was based on the length and structure of the duodenum, jejunum, and ileum. Table 6 shows the length and structure of laying hens' intestines when their diet was supplemented with lysozyme.

**Table 6:** Effect of dietary supplementation with lysozyme on intestinal morphology.

**Item**	**Level of lysozyme**					**SEM**	***p*** **-value**		
	**0**	**100**	**200**	**300**	**400**		**ANOVA**	**Linear**	**Quadratic**
**Duodenum**									
Villus height (μm)	826.27	835.28	817.78	838.54	836.21	11.66	0.983	0.785	0.953
Crypt depth (μm)	110.94	103.22	97.20	96.98	99.94	2.35	0.315	0.089	0.087
Villus crypt ratio	7.59	8.10	8.48	8.68	8.46	0.17	0.249	0.045	0.063
**Jejunum**									
Villus height (μm)	574.90	580.52	583.48	545.63	643.38	12.66	0.171	0.262	0.214
Crypt depth (μm)	100.52	106.34	86.81	75.95	82.61	4.19	0.112	0.023	0.074
Villus crypt ratio	5.80^b^	5.88^b^	6.84^a^	7.41^a^	7.91^a^	0.26	0.019	0.001	0.003
**Ileum**									
Villus height (μm)	588.10	607.27	574.39	561.80	590.32	12.91	0.861	0.661	0.856
Crypt depth (μm)	80.90	77.38	70.99	69.69	73.10	1.74	0.220	0.056	0.066
Villus crypt ratio	7.30	7.91	8.17	8.09	8.13	0.18	0.561	0.158	0.230

a, b Mean that there were significant difference between groups.

The results in Table 6 indicated that dietary supplementation with lysozyme had no significant effect on the villus height, crypt depth, and villus crypt ratio in the duodenum and ileum (*P* > 0.05), but a linear effect (*P* = 0.045) on the villus crypt ratio in the duodenum was noted with increasing dietary lysozyme concentration. In the jejunum, there was no difference in the villus height and crypt depth (*P* > 0.05), but linear (*P* = 0.001) and quadratic (*P* = 0.003) effects were observed on the villus crypt ratio in the 200, 300, and 400 mg/kg lysozyme groups compared with the control group.

### 3.6. Nutrient digestibility

As the nutrient digestibility of laying hens shows their feed utilization, in this current study, the apparent nutrient digestibility of laying hens was analyzed when their diet was supplemented with lysozyme. Table 7 presents the results of lysozyme's effect on nutrient retention of laying hens.

**Table 7:** Effect of dietary supplementation with lysozyme on nutrient retention of laying hens.

**Item**	**Level of lysozyme**					**SEM**	***p*** **-value**		
	**0**	**100**	**200**	**300**	**400**		**ANOVA**	**Linear**	**Quadratic**
Crude Protein (%)	34.04^b^	39.96^b^	35.82^b^	47.94^a^	35.48^b^	1.34	0.002	0.258	0.039
Organic matter (%)	72.01^b^	74.80^b^	74.44^b^	79.03^a^	72.28^b^	0.60	< 0.001	0.271	0.006
Dry matter (%)	64.74^c^	68.12^b^	67.88^ab^	73.18^a^	65.07^ab^	0.71	< 0.001	0.265	0.004
Total energy (%)	72.86^b^	76.04^b^	75.44^b^	80.55^a^	73.65^b^	0.66	< 0.001	0.198	0.009

a, b Mean that there were significant difference between groups.

Table 7 shows that the laying hens' apparent retention of organic matter, dry matter, and total energy was quadratically increased when their diet was supplemented with lysozyme (*P* < 0.05) in comparison with the control group. The highest value was observed with the 300 mg/kg lysozyme group which could also increase (*P* < 0.05) laying hens' apparent retention of crude protein quadratically.

## 4. Discussion

In laying hens, feeding is one of the keys to successful egg production performance and influences the benefits of farmers. Our research showed that all levels of lysozyme had no significant influence on laying rate, egg mass, soft and broken egg rate, feed intake, FCR, and egg weight (*P* > 0.05) but significantly decreased the sand-shelled egg during 4–8 weeks when compared with the control group (*P* < 0.05). Similarly, some studies reported that dietary supplementation with lysozyme could improve the growth performance of chicken and weaning piglets (16, 21, 23). However, other investigations conducted on broiler chicken had shown that dietary supplementation with lysozyme did not increase the growth performance (17, 24, 25), which supports the results of our current study. These differences should be influenced by several aspects including the lysozyme level, enzyme activity, lysozyme production technology, nutritive value of basal diet, management, and the farm's environment (25, 26). This result illustrated that dietary supplementation with lysozyme impacted the laying performance positively by improving the egg's external quality (27). The decrease in sand-shelled eggs by lysozyme should be influenced by its action on defects of eggshell glands and calcium deposits which can often be attributed to disturbance during the shell calcification in the oviduct (28–30). The positive aspect of lysozyme on egg external quality should be attributable to the improvement of intestinal morphology and nutrient retention. These improvements may be due to the enhanced intestinal integrity for improved absorptive capacity of nutrients (31, 32). In our current research, the intestinal morphology in the jejunum and the apparent nutrient retention were improved with lysozyme supplementation, but we did not measure the apparent nutrient retention of calcium and total phosphorus. However, because the information available on the effects of supplementation with lysozyme on laying hens at the late phase is scarce, more studies are necessary to comprehend how lysozyme reduces the rate of sand-shelled eggs of laying hens.

There was no difference between the lysozyme group and control group on the quality of qualified eggs, which shows that dietary supplementation with lysozyme had no adverse effect on eggs' quality, and it can respond to the need for egg quality production. Many factors can influence the egg quality of hens including genetics, environment, behaviors, body weight, handling eggs during collection, and the hen's growth stage during the laying period (27, 33, 34). The intestinal barrier function of laying hens may be affected by the late phase, resulting in reduced digestive enzyme activity and nutrient deposition, which negatively affects egg quality after the peak laying period (35). However, dietary lysozyme supplementation is safe for egg quality. There was no significant effect on biochemical parameters, which showed that the liver and kidney (health) of laying hens were not affected by lysozyme, indicating its safety for laying hens breeding. This result is similar to previous studies which reported that lysozyme is safe and without toxicity (21, 36). Furthermore, ALT and AST are the enzymes that characterize liver injury (37). Our research showed that the AST level quadratically decreased with the increasing level of lysozyme, which also indicates improved overall health (37).

In animals, the oxidation of dietary amino acids is characterized by blood urea nitrogen (BUN) (38, 39). Our research showed that lysozymes did not significantly change the BUN concentration in plasma, which shows the proper utilization of dietary amino acids by laying hens. However, it was found that on the 35th day, 120 mg/kg lysozyme could improve the total protein and globulin in the serum of broiler chickens (21). In contrast, it has been reported that replacing colistin sulfate with lysozyme (100 mg/kg) reduced serum concentrations of total protein and globulin (40). As the redox balance is essential to animal health, it may influence the antioxidant capacity and free radical generation (41). Our results showed that lysozyme did not significantly influence the laying hens' antioxidant capacity, which is relative to other research studies (42). Similarly, dietary supplementation with exogenous 90 mg/kg lysozyme stimulated ileum SOD1 and GSH-Px gene expression, resulting in a significant increase in intestinal detoxification status against various xenobiotics (21). Lysozyme is an important non-specific immune-modulating factor (43, 44). In laying hens, immune organs and immune cells usually are the components of non-specific immunity. Humoral immunity is provided by lymphocytes; B cells and T cells are responsible for cellular immunity. Many times, an examination of immune function is conducted on basic IgG and IgM level indices (45). IgG is very important in fighting bacterial and viral infection, while IgM is considered a crucial immunoglobulin that works as an anti-inflammatory response by causing other cells' immune systems to destroy foreign substances. The increase of IgM in the plasma of laying hens indicates a better immune status (45, 46). In this study, compared with the control group, no significant effect of lysozyme on IgA, IgM, and IgG was observed (both linear and quadratic *P* > 0.05), indicating that dietary supplementation with lysozyme had no negative effect on immune function. As central to the development and effector activities of immune responses, cytokines are important components of the immune and inflammatory responses (47). Our results showed that with the increasing lysozyme level, the concentration of IL-2 in plasma decreased linearly and quadratically, especially the 400 mg/kg had the lowest IL-2 level, which indicates that lysozyme consumption decreased pro-inflammatory cytokines of laying hens and improved immune efficiency. Our results did not show a significant difference for other cytokines (IL-4, TNF-α, IFN-γ, and IL-6), which is relatively similar to previous research (16, 40). Consistently, the piglets receiving the feed supplemented with lysozyme decreased TNF-α when they were exposed to a challenging environment (20, 39). These different results are likely due to our laying hens raising in good management and low-stressed condition.

The small intestine is a vital organ for maintaining digestive, endocrine, metabolic, and immune functions in animals (48). This research indicated that supplementation with 200, 300, and 400 mg/kg lysozyme improved significantly the laying hens' villus crypt ratio in the jejunum (both linear and quadratic *P* < 0.05). This result is similar to other authors who reported that supplementation with lysozyme could improve the intestinal morphology of chicken (21, 25, 36, 49). Lysozyme did not significantly influence the structure of the ileum and duodenum. These results are in agreement with the research conducted on pigs (22, 40, 49). As the jejunum is in the middle of the small intestine and is the primary site for the digestion and absorbing process, this may explain why laying hens fed with a lysozyme diet have the greatest influence on the jejunum morphology. Lysozyme could quadratically improve the apparent retention of crude protein, total energy, and organic and dry matter, which indicates better nutrition utilization by feeding laying hens with a lysozyme diet. Consistently, it is reported that dietary supplementation with lysozyme improves nutrient absorption in chickens and pigs (50–52).

## 5. Conclusion

Dietary supplementation with lysozyme could increase laying hens' production performance by lowering the sand-shelled egg rate and plasma IL-2 level and promoting the intestinal morphology of the jejunum, immune efficiency, and nutrient absorption. The supplementation with 200 to 300 mg/kg lysozyme should be recommended to farmers as the appropriate lysozyme for laying hens breeding. This level of lysozyme could replace the antibiotic use in laying hens feeding without more effect on the famers' profitability.

## Data availability statement

The original contributions presented in the study are included in the article/supplementary material, further inquiries can be directed to the corresponding author.

## Ethics statement

The animal study was approved by Maoming Branch, Guangdong Laboratory for Lingnan Modern Agriculture, Guangdong Provincial Key Laboratory of Animal Nutrition Control, National Engineering Research Center for Breeding Swine Industry, College of Animal Science, South China Agricultural University, Guangzhou, China. The study was conducted in accordance with the local legislation and institutional requirements.

## Author contributions

DS: Conceptualization, Data curation, Formal analysis, Methodology, Software, Writing—original draft. ZXia: Conceptualization, Data curation, Formal analysis, Methodology, Writing—original draft. CW: Data curation, Writing—review and editing. PL: Data curation, Writing—review and editing. ZXin: Data curation, Writing—review and editing. DG: Data curation, Writing—review and editing. SY: Data curation, Writing—review and editing. KY: Writing—review and editing. FZ: Writing—review and editing, Data curation. LZ: Data curation, Writing—review and editing. SHe: Data curation, Writing—review and editing. SHa: Writing—review and editing. SZ: Writing—review and editing. BD: Writing—review and editing, Supervision.
